# Adsorption of the rhNGF Protein on Polypropylene with Different Grades of Copolymerization

**DOI:** 10.3390/ma16052076

**Published:** 2023-03-03

**Authors:** Paolo Canepa, Claudio Canale, Ornella Cavalleri, Giovanni Marletta, Grazia M. L. Messina, Massimo Messori, Rubina Novelli, Simone Luca Mattioli, Lucia Apparente, Nicola Detta, Tiziana Romeo, Marcello Allegretti

**Affiliations:** 1 Dipartimento di Fisica, Università di Genova, Via Dodecaneso 33, 16146 Genova, Italy; 2 Laboratory for Molecular Surface and Nanotechnology (LAMSUN), Dipartimento di Scienze Chimiche, Università di Catania and CSGI, Viale A. Doria 6, 95125 Catania, Italy; 3 Department of Applied Science and Technology, Politecnico di Torino, Corso Duca degli Abruzzi 24, 10129 Torino, Italy; 4 Research & Early Development, Dompè Farmaceutici S.p.A., Via Santa Lucia 6, 20122 Milano, Italy; 5 Research & Early Development, Dompè Farmaceutici S.p.A., Via De Amicis 95, 80131 Napoli, Italy; 6 Research & Early Development, Dompè Farmaceutici S.p.A., Loc. Campo di Pile, 67100 L’Aquila, Italy

**Keywords:** rhNGF, protein adsorption, polypropylene, QCM-D, XPS, DSC, AFM, contact angle

## Abstract

The surface properties of drug containers should reduce the adsorption of the drug and avoid packaging surface/drug interactions, especially in the case of biologically-derived products. Here, we developed a multi-technique approach that combined Differential Scanning Calorimetry (DSC), Atomic Force Microscopy (AFM), Contact Angle (CA), Quartz Crystal Microbalance with Dissipation monitoring (QCM-D), and X-ray Photoemission Spectroscopy (XPS) to investigate the interactions of rhNGF on different pharma grade polymeric materials. Polypropylene (PP)/polyethylene (PE) copolymers and PP homopolymers, both as spin-coated films and injected molded samples, were evaluated for their degree of crystallinity and adsorption of protein. Our analyses showed that copolymers are characterized by a lower degree of crystallinity and lower roughness compared to PP homopolymers. In line with this, PP/PE copolymers also show higher contact angle values, indicating a lower surface wettability for the rhNGF solution on copolymers than PP homopolymers. Thus, we demonstrated that the chemical composition of the polymeric material and, in turn, its surface roughness determine the interaction with the protein and identified that copolymers may offer an advantage in terms of protein interaction/adsorption. The combined QCM-D and XPS data indicated that protein adsorption is a self-limiting process that passivates the surface after the deposition of roughly one molecular layer, preventing any further protein adsorption in the long term.

## 1. Introduction

The development of drugs based on the use of biologically-derived products has rapidly advanced over the past 20 years [1,2,3], outpacing that of traditional, chemically-synthesized, small-molecule drugs [4,5,6]. In this context, the investigation of the interactions between protein-based agents and solid surfaces has become increasingly important in the pharmaceutical sector, as it is fundamental to understand how packaging materials can interact with the biologicals that they contain [7,8,9,10]. The selection of the optimum packaging material is crucial, in fact, for maintaining the structural integrity and functional effectiveness of biological products during drug production, transport, and storage processes [11], thus ensuring the quality of the pharmaceutical product and patient safety [7].

The Nerve Growth Factor (NGF) is a complex protein that promotes the survival, maintenance, proliferation, and activity of neuronal cells, as well as the differentiation of premature neuronal cells into post-mitotic mature neurons [12,13]. Due to its biological functions, NGF has increasingly attracted the interest of the scientific community and has been considered as a potential therapeutic approach for the treatment of multiple diseases [14,15]. So far, a form of rhNGF (recombinant human Nerve Growth Factor) is used in an ophthalmic solution filled in glass vials, authorized in several countries for the treatment of patients with a rare corneal disease [16,17]. As with every other biological product, rhNGF can interact non-specifically with the surface of a variety of materials, such as glass and plastics, with which it may come in contact during production, storage or administration processes. In this context, primary packaging of the final product is of utmost importance as it is in direct contact with the drug product during long-term shelf-life, and it should then be sufficiently inert not to cause any alteration in the product composition. For liquid drug forms, high quality glass or plastic materials are typically used as primary packaging for the finished product [18,19]. However, although chemically inert, protective and highly transparent, glass is fragile and sometime difficult to handle. On the other hand, plastics are flexible and low weight, but some drugs could react more easily with the container, thus altering their quality.

Polypropylene (PP)-based materials have been approved by Pharmacopoeias for pharmaceutical primary packaging and are commonly used. Different types of PPs are available on the market, displaying different crystallinity and degrees of copolymerization with polyethylene (PE), which can lead not only to different surface roughness and mechanical properties of the material, but also to different physicochemical surface properties that can in turn influence the adsorption of the biomolecules. To investigate the interaction between biological macromolecules and surfaces, different experimental and computational approaches have been proposed in the literature [20,21,22,23,24]. They include mass sensitive methods such as Quartz Crystal Microbalance (QCM) [25,26] or nanomechanical resonators [27], as well as optical methods, from surface plasmon resonance (SPR) [26,28] to spectroscopic ellipsometry [29,30]. Spectroscopic methods can be advantageously coupled to high resolution microscopic investigations for the analysis of ultrathin molecular layers [31]. Of course, the more complex the structural and physicochemical properties of the surface, the more challenging is the accurate evaluation of molecule/surface interaction. Indeed, the quantification of protein adsorption on polymer surfaces having the same properties as that employed in packaging is a quite challenging task, not easily achievable with the use of only a single analytical technique. Thus, a reliable characterization can derive only from the correlation of complementary data from different surface sensitive methods.

In the present study, we evaluate the interactions between liquid solution of rhNGF and some grades of PP-based plastic materials (polypropylene, PP) that are usually used to produce a multidose packaging of ophthalmic drug product (i.e., squeeze containers for eyedrops). To this end, we developed a sophisticated multi-technique approach that allowed us to quantify, directly and indirectly, the adsorption of the pharmacologically relevant protein to different polymeric materials. In particular, we analyzed the intrinsic protein adsorption properties of different PP/PE and PP materials, assessed the degree of crystallinity of PP/PE copolymers and PP homopolymers, finally evaluating the short- and long-term adsorption of protein on the inner surface of some commercially used PP/PE containers.

## 2. Materials and Methods

### 2.1. Materials

In this study, we used the pharmaceutical grade polymeric materials listed in Table 1, already approved for the manufacture of drug primary containers. We investigated the properties of PP/PE copolymers of the Bormed^TM^ series from Borealis (Vienna, Austria), in particular, the Bormed^TM^ RD808CF, Bormed^TM^ SB815MO, and Bormed^TM^ SC876CF, and two PP homopolymers, Bormed^TM^ HD810MO and Purell HP372P from LyondellBasell (Rotterdam, The Netherlands), hereafter referred to as PP1-PP5 as reported in Table 1. We purchase the polymers in solid form, after injection molding, in the form of lozenges.

Spin-coated polymer films were deposited on QCM sensors for QCM-D analysis and on silicon wafers for AFM analysis according to the following procedure.

SiO_2_-coated sensor crystals for QCM-D analysis (Q-Sense AB, Biolin Scientific, Gothenburg, Sweden) and silicon wafers (<100>, from Siegert Wafer GmbH, Charlottenburger, Germany) were used as substrates. The surfaces were cleaned individually by 20 min of UV-Ozone treatment, λ_exc_ = 185 nm and 254 nm (Jelight Company, Irvine, CA, USA), then they were rinsed by milli-Q ultrapure water and gentle dried with nitrogen flow. Silicon wafers were atomically flat and SiO_2_-coated sensors had a roughness lower than 1.3 ± 0.1 nm.

Based on literature data, 2% solutions (by weight) of polymeric materials were used to deposit thin polymer films on the substrates. In particular, decaline (from Sigma-Aldrich, Darmstadt, Germany) was used as a solvent, at a temperature above 160 °C to obtain a complete polymer dissolution, in 20 min on sand bath. After the solution was obtained, the substrates and the spin coater chuck were heated at about 160 °C to ensure the maintenance of liquid state of the solution, in order to obtain, finally, uniformly spun films on the substrates. Spin rates used to deposit the polymer solution were: 1000 rpm for 10 s, 2000 rpm for 10 s and 3500 rpm for 60 s. The solution volume used ranged between 80 and 100 μL. After coating, the samples were let to cool down slowly for 12 h.

Film thicknesses, measured with a profilometer (Alpha Step, KLA Instruments, Milpitas, CA, USA), are reported in Table 2.

Injection molded specimens were used for Differential Scanning Calorimetry (DSC), Contact Angle (CA), and X-ray Photoelectron Spectroscopy (XPS) analysis.

The protein used in the present study corresponds to a liquid form of recombinant human Nerve Growth Factor (rhNGF) by Dompé supplier (Biotech Facility-L’Aquila, Italy), characterized by a rhNGF technical batch purity of 87.6% measured by reverse phase HPLC.

Protein adsorption experiments were carried out using a 0.8 µM rhNGF solution, prepared by Dompé Technological Lab Department starting from rhNGF Reference Standard at a concentration of 33 µM.

### 2.2. Atomic Force Microscopy (AFM) Analysis

Atomic Force Microscopy (AFM) measurements were carried out in Tapping Mode (TM) by using a Nanoscope IV-MultiMode AFM (Digital Instruments, Santa Barbara, CA, USA). The device was equipped with a <J> scanner calibrated using grating manufacturers. Images were recorded at a scan rate of 1 Hz and 512 × 512 pixels per image (i.e., in high resolution conditions) by using n-doped silicon cantilevers with a nominal force constant of 40 N/m and a resonant frequency of 300 kHz. The amplitude setpoint was set at about 75% of the free oscillation amplitude to minimize the tip/sample interaction. Image analysis was carried out using DI software, version 5.30. The images were flattened to remove background slopes.

Sample roughness was measured on 1 × 1 μm^2^ TM images for a minimum of six separate zones for each sample obtained from different regions. Roughness is expressed in terms of Ra, the arithmetic average of the absolute values of the surface height deviations measured from the mean plane within the cursor box using:

(1) $$R_{a}=\frac{1}{n}\overset{n}{\underset{j=1}{\sum }}\left|Z_{j}\right|$$

### 2.3. Quartz Crystal Microbalance with Dissipation (QCM-D) Monitoring

The adsorption kinetics measurements were performed by using a Quartz Crystal Microbalance with Dissipation Monitoring (QCM-D) instrument (Q-Sense AB, Biolin Scientific, Gothenburg, Sweden). The simultaneous measurement of frequency, f, and energy dissipation, D, was performed for the fundamental resonance frequency (*n* = 1, i.e., f = 5 MHz) and the overtones (*n* = 3, 5, 7, and 9 corresponding to f = 15, 25, 35, and 45 MHz, respectively). The resolution in f and D was ±0.1 Hz and 1 × 10^−7^, respectively. Each QCM-D experiment started with the sensor running on PBS (outgassed with 30 min sonication), then by adding the rhNGF solution and, after 60 min, exchanging the solution being measured with PBS to check both stability and desorption of the adsorbed layer. All the experiments were performed in PBS at 25 °C and the flow rate was 150 μL min^−1^.

### 2.4. Thermal Analysis

Measurement of thermal properties (melting enthalpy, ΔH_m_, melting temperature, T_m_, and degree of crystallinity, X_c_) of PPs was performed by Differential Scanning Calorimetric (DSC) Analysis. DSC analysis was carried out with a DSC TA 2010 instrument (TA Instruments, New Castle, DE, USA), using (8 ± 1) mg of sample and operating by heating from 0 °C to 200 °C at a heating rate of 20 °C/min. The chamber was purged by nitrogen at 50 mL/min. Samples were obtained by manual cutting from injection molded specimens. Two samples for each material were tested. The degree of crystallization was calculated considering the value of 208 J/g for the 100% crystalline PP melting enthalpy [32,33].

### 2.5. Static Contact Angle (CA) Analysis

The contact angle measurements were performed using a Dataphysics—Contact Angle System OCA 200 instrument (DataPhysics Instruments GmbH, Filderstadt, Germany), with an electronic control software with automatic alignment. Ten contact angle measurements have been made for each material and then the mean value and the standard deviation were calculated. To minimize the error induced by the different roughness of the sample surfaces generated by the injection molding process, five measurements were taken on the front face and five measurements on the back face of each sample. For the rhNGF solution, a total of 10 drops of 3 µL each were deposited on each PP sample.

### 2.6. X-ray Photoelectron Spectroscopy (XPS) Analysis

XPS measurements were carried out using a PHI 5600 Multi-Technique apparatus (Chanhassen, MN, USA), equipped with an X-ray Al-monochromatized source (hυ = 1486.6 eV). A neutralizer (low energy electron flood gun) was used during measurements to avoid sample charging [34]. Wide scan spectra were acquired using a pass energy of 187.85 eV, while high-resolution spectra of the C1s and N1s core level regions were acquired using a pass energy of 46.95 eV. The binding energy (BE) scale was calibrated by setting the C1s component of adventitious carbon at a binding energy of 284.8 eV. Spectra analysis was performed with CasaXPS software (v2.3.25PR1.0, Casa Software Ltd., Teignmouth, UK); after a Shirley background subtraction, 30% gaussian Voigt functions were used for spectra deconvolution.

## 3. Results

### 3.1. Protein Adsorption Analysis on PP Model Surfaces

**Figure 1:**
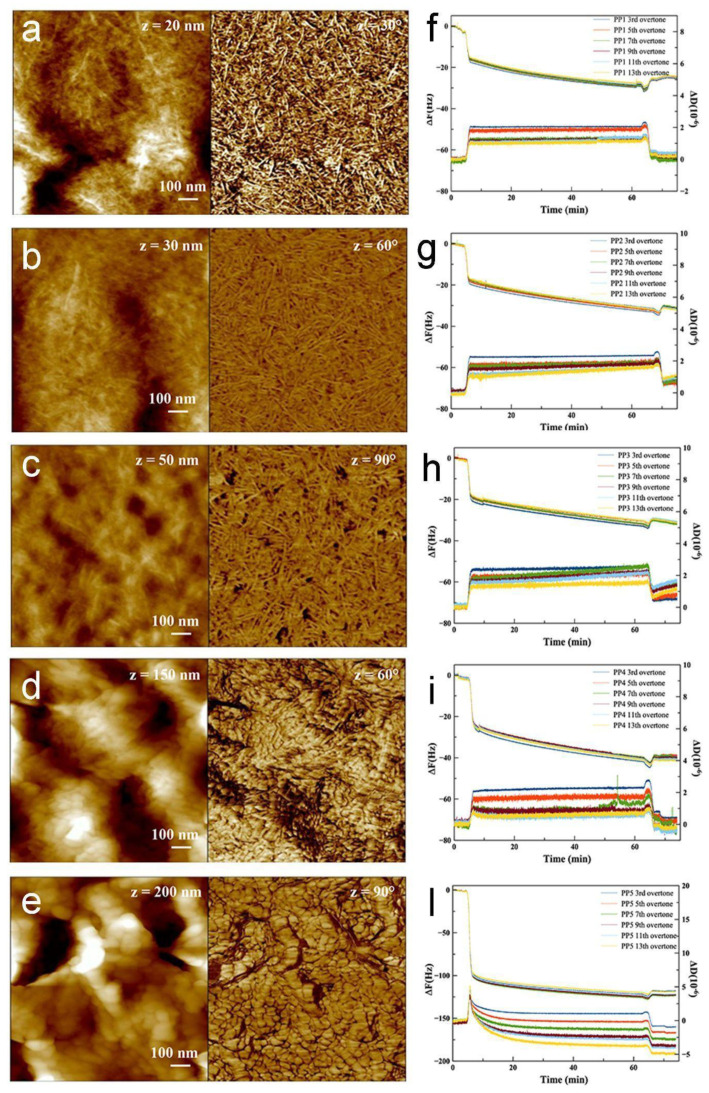
TM AFM images (height left, phase right) of (**a**) PP1, (**b**) PP2, (**c**) PP3, (**d**) PP4, and (**e**) PP5 films. QCM-D graphs relative to rhNGF adsorption onto (**f**) PP1, (**g**) PP2, (**h**) PP3, (**i**) PP4, and (**l**) PP5 films.

In order to assess the intrinsic properties of PP homopolymers and PP/PE copolymers in terms of protein adsorption, we exploited QCM-D, a highly sensitive method for quantifying molecular deposition on surfaces. To perform QCM-D analysis, we prepared thin polymeric films by spin-coating directly on the silicon QCM-D sensor surface. In parallel, an AFM investigation was performed to correlate protein adsorption with polymer film morphology. We note that, with the roughness of the substrates (both QCM sensors and silicon wafers) being much lower than the polymer film thickness, the surface morphology of the polymer films is not affected by the substrates, and the AFM images reported in Figure 1 are representative of the polymer films deposited on both substrates.

Figure 1a–e reports the morphology at the nanoscale, both in height and phase images, for the three PP/PE copolymer films (Figure 1a–c) and for the two PP homopolymer films (Figure 1d–e). For the three copolymer samples, phase images (right images) highlight a very dense networks of lamellae, which have roughly the same physical dimensions in all cases; both the substrate coverage and the roughness appear substantially uniform. On the other hand, the two homopolymer samples displayed a different morphology (panels of Figure 1d–e), showing non-lamellar components, more resembling flakes, and, globally, a higher roughness compared to the three copolymer samples. Average roughness values measured on 1 × 1 μm^2^ images are reported in Table 3.

Figure 1f–l reports the adsorption kinetics of rhNGF on the polymer surfaces, which was measured by in situ QCM-D. The rhNFG mass uptake for each sample was evaluated from the shift of the sensor resonance frequency (Δf), which was calculated as the difference between frequency values before protein solution injections and after buffer rinsing, while the changes in dissipation value (ΔD) provided information on the viscoelastic response of the adsorbed rhNGF layer. As far as the measured dissipation value was lower than 5.0 × 10^−6^, it was possible to use the Sauerbrey equation [35] to provide the mass uptake Δ*m*:

(2) $$\Delta m=-\left(C/n\right)\cdot \Delta f$$

where ∆f represents the decrease in resonant frequency, *C* is a constant depending on intrinsic properties of the quartz slab (in our case *C* = 17.7 ng/cm^2^ Hz^−1^ at f = 5 MHz), and *n* = 1, 3, 5… is the overtone number. The surface molecular density was estimated from the deposited mass given the molecular weight of rhNGF.

The calculated mass uptake and molecular density values are reported in Table 3. Mass uptake values ranging from 430 ng/cm^2^ to 514 ng/cm^2^ and, in turn, values of the adsorbed rhNGF molecules in the order of (1.0–1.2) × 10^13^ molecules/cm^2^ were obtained for the three copolymer substrates. The calculated surface molecular densities suggest that roughly one molecular monolayer was adsorbed by the polymeric surface [36]. This finding, supported by the QCM-D trend, suggests a self-limiting process induced by the passivation of the surface after the deposition of roughly one molecular layer. Notably, the rhNGF mass uptake was remarkably higher for the two homopolymer samples, indicating that the higher surface roughness (i.e., the larger adsorbing area, measured by AFM), together with the higher wettability, inferred from contact angle measurements on injection molded samples (see Section 3.2), could account for the higher mass uptake observed on the homopolymer samples compared to the copolymer ones.

It is worthwhile to mention that since each harmonic frequency resonance penetrates to a different distance from the sensor surface, different harmonics sample different thicknesses of the deposited layer and overlaying fluid. In particular, the higher harmonics probe sample layers relatively close to the quartz sensor surface, whereas lower harmonics extend beyond the adsorbed layer into the bulk solution. Accordingly, the spreading of the traces of different harmonics is diagnostic of the inhomogeneity of the deposited layers (along the z-direction) and, in turn, of the “softness” of the adsorbed layer. In view of this, it is noteworthy that, as shown in Figure 1f–h, the recorded overtones are very close to each other for the copolymer substrates, suggesting that the adsorbed rhNGF layer is indeed rigid, in agreement with the very low value of dissipation. Conversely, a higher overtone spreading is observed in the case of homopolymers (Figure 1i,l), especially for PP5. This finding, together with the higher values of mass uptake, suggests that the homopolymer surfaces adsorb thicker, somehow softer, and more hydrated protein films.

Additional information can be obtained from the D-f plots (Figure S1, Table S1), reporting the correlation between dissipation (ΔD) and frequency (Δf) values. As reported in the Supporting Information, the D-f plots highlight the occurrence of two adsorption kinetic regimes and allow the evaluation of the sticking efficiency of rhNGF to the surface and the convection-driven protein mass transport/binding at the surfaces [37,38].

Overall, the QCM-D analysis of the intrinsic rhNGF adsorption on polymers indicates that protein adsorption on the copolymers (PP1–PP3) is lower than that on the homopolymer films (PP4, PP5). QCM-D results on PP films can serve as a guide for the following analysis of injection molded specimens, whose properties are expected to be closely related to those of commercially used squeeze containers for eyedrops.

### 3.2. Characterization of PP Injection Molded Specimens

The thermal properties of injection molded polymer specimens were investigated by Differential Scanning Calorimetric (DSC) and correlated with surface roughness and wettability data. The melting temperature, T_m_, melting enthalpy, ΔH_m_, degree of crystallinity, X_c_, roughness, R_a_, and contact angle values of PP1–PP5 specimens are reported in Table 4.

As expected, copolymers (PP1–PP3) have lower melting temperatures (both onset and peak) and degree of crystallinity compared to homopolymers (PP4 and PP5). The tested PPs can be ordered based on their degree of crystallinity (proportional to the respective melting enthalpy) as shown below (from left to right, from the most to the least crystalline):

PP4 > PP5 > PP1 >PP2 >PP3.

As expected, PP homopolymers have a higher crystallinity (around 40%) while the degree of crystallinity of PP/PE copolymers is in the 20–30% range. The lower crystallinity of copolymers results in a higher ductility of these materials and makes them more suitable for packaging production than homopolymers.

The contact angle values of the rhNGF solution on the PP substrates can be considered as an indication of the affinity/interaction between solid and liquid pair: the lower the contact angle, the higher the affinity/interaction. Contact angle data indicate a correlation with chemical composition and structure (homopolymer or copolymer and comonomer content) which are, in turn, related to the degree of crystallinity and surface roughness.

**Figure 2:**
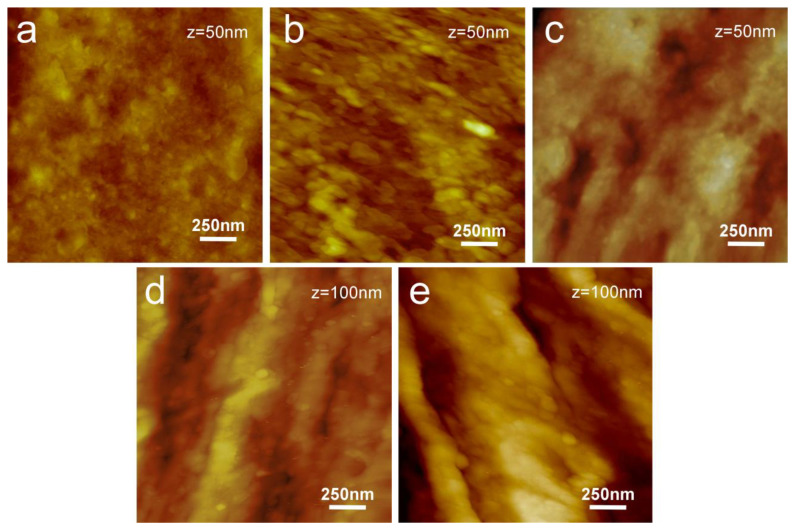
TM AFM height images of (**a**) PP1, (**b**) PP2, (**c**) PP3, (**d**) PP4, and (**e**) PP5 samples.

The roughness of the injection molded specimens was evaluated by AFM. Figure 2 shows the typical morphology of the three PP/PE copolymers and the two PP homopolymer samples. For each sample, three different areas were analyzed and the average R_a_ value was calculated from the analysis of the three images. To obtain a statistically relevant result, for each PP material, an average Ra value was calculated from the analysis of ten injected samples.

AFM analysis shows some morphological differences between PP/PE copolymers and PP homopolymers. In particular, the corrugations on the PP homopolymer surfaces are higher than those on PP/PE copolymers. This aspect is reflected in the roughness that is slightly higher for the PP homopolymers.

Contact angle measurements were performed to assess the wettability of the different homo- and copolymers. Average contact angle and standard deviation values recorded for the PPs are reported in Table 4. The contact angle values can be considered as an indication of the affinity/interaction between solid and liquid: the lower the contact angle, the higher the affinity/interaction. Data reported in Table 4 show the highest values of contact angle for copolymer samples (PP1, PP2, and PP3). Contrary to expectation, contact angle data seem to be not strictly correlated with the degree of crystallinity. From this point of view, the different chemical composition and structure (homopolymer or copolymer and comonomer content) of PP seem to be the more relevant factor for establishing the interaction degree with the rhNGF. The combined DSC, AFM, and CA analysis on injected molded specimens indicates a correlation between chemical composition and structure (homopolymer or copolymer and comonomer content), degree of crystallinity, roughness, and wettability: copolymers, characterized by a lower degree of crystallinity and lower roughness, show higher contact angle values and are, therefore, better suited for packaging production since they are expected to adsorb rhNGF to a lesser extent compared to homopolymers.

The combined analyses on PP spin-coated films and injected molded specimens indicate that copolymers have reduced interaction with the rhNGF solution and reduced rhNGF adsorption. Therefore, we selected copolymers, in particular PP3, the material currently used to produce squeeze containers for ophthalmic solutions, to investigate the long-term interaction between the rhNGF solution and the inner surface of PP3 commercial squeeze containers. As discussed in the following, XPS analysis confirms that protein adsorption is a very fast process that occurs within the first minutes. Therefore, the similar short-term adsorption behavior of the three copolymers suggests a similar long-term behavior to be expected for the other copolymers, PP1 and PP2.

### 3.3. Measurement of the Adsorption of rhNGF on the Surface of PP3 Bottle by X-ray Photoelectron Spectroscopy (XPS)

Since QCM-D cannot be applied to thick bottle walls, XPS was chosen to evaluate the adsorption of protein on PP3 bottles (an AFM image of internal bottle surface is reported in Figure S2). Different from QCM-D, XPS cannot be used for in situ real-time monitoring of protein adsorption, but it does allow the evaluation of molecular adsorption over an extended range of exposure times, from a few seconds up to months (i.e., long exposures comparable to common storage time for commercial purposes). The influence of the solution storage temperature can also be addressed by XPS analysis.

**Figure 3:**
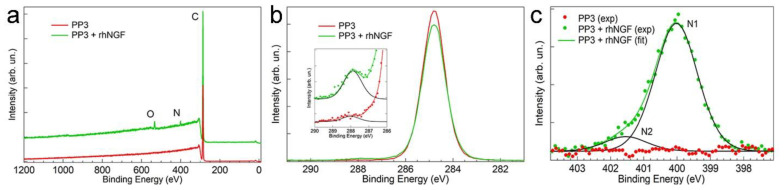
(**a**) XPS survey spectra, (**b**) C1s core level region, and (**c**) N1s core level region of PP3 before (red curves) and after (green curves) 30 s incubation in the rhNGF solution.

Figure 3a shows the XPS survey spectra acquired on the inner side of the PP3 bottle before (red curve) and after (green curve) 30 s exposure to the protein solution. The occurrence of protein adsorption is easily inferred from the presence of the N1s and O1s signals not detected on the bare sample.

To obtain a deeper insight into protein adsorption, we acquired high resolution XPS spectra of selected core level regions. The C1s and N1s signals of a PP3 bottle before (red curve) and after (green curve) 30 s exposure to the protein solution are reported in Figure 3b,c, respectively. Interestingly, incubation in rhNGF significantly modifies the C1s signal in the spectral region typical of the COOH group. As can be observed in the inset of Figure 3b, the carboxylic-related C1s component at a BE of (288.0 ± 0.2) eV [39,40] shows a neat intensity increase upon incubation in the rhNGF solution, a signature of the protein adsorption. As concerns the second molecular marker, the N1s signal, typical N1s XPS spectra of a PP3 bottle before (red curve) and after (green curve) exposure to the protein solution is reported in Figure 3c. As expected, no detectable N1s signal is observed on the bare PP3 bottle. The deconvolution of the N1s signal acquired after exposure to the protein solution shows the presence of two N1s components, N1 at a BE of (399.8 ± 0.2) eV and N2 at a BE of (401.5 ± 0.2) eV, that can be assigned to the NH_2_ and NH_3_^+^ groups of the protein, respectively [41,42].

The N1s/C1s intensity ratio was chosen to monitor rhNGF adsorption. For a comparative quantification of the protein adsorption, we measured the N1s and C1s signals as a function of the exposure time (30 s, 7 days, and 30 days) at both 4 °C and 25 °C. The N1s/C1s intensity ratio values calculated for the different exposure conditions are reported in Table 5.

The data confirm that adsorption of rhNGF is a very rapid process that occurs within the first few seconds of the interaction of the protein solution with the bottle surface. N1s/C1s values measured after 30 s remain substantially constant over an extended period (30 days), indicating an adsorption/passivation mechanism.

Storage temperature variation within the 4–25 °C range also does not result in any significant change in the rhNGF adsorption. Data indicate that refrigerated storage does not result in significant advantages in terms of reducing protein adsorption on the surface of the container. Therefore, the drug can be stored at room temperature with reduced delivery and storage costs. However, storage at lower temperatures can reduce possible unwanted phenomena such as protein denaturation and aggregation. Further studies are necessary to investigate these other aspects.

## 4. Conclusions

A multi-technique approach has been used to characterize the adsorption of rhNGF, the active principle of an ophthalmic drug, on different pharma-grade PP/PE copolymers and PP homopolymers. On both PP/PE and PP films, QCM-D data indicated the occurrence of a two-step adsorption process, with a first rapid protein deposition step (ca 80% of the total mass deposited within the first few minutes) that is followed by a slower adsorption process, and the calculated surface density of the molecule suggests roughly the formation of one molecular monolayer. In addition, QCM-D analysis indicated that the chemical composition of the polymer films greatly influences protein adsorption: PP/PE copolymers are found to adsorb lower amounts of protein compared to PP homopolymers. The higher adsorption on homopolymer films can be partly related to their higher surface roughness as results from AFM analysis. These findings are in line with the contact angle analysis on injection molded specimens, which indicated a higher surface wettability for the rhNGF solution on PP homopolymers. Moreover, in this case, the higher wettability can be associated with the higher surface roughness of homopolymer compared to copolymer samples. Finally, XPS analysis was exploited to investigate the effects of long-term interaction between the rhNGF solution and the surface of PP/PE copolymers, a typical material used in some commercial squeeze containers for eyedrops.

To summarize, we demonstrated that the chemical composition of the polymer material and, in turn, its surface roughness may influence the interaction with some biological solutions. More specifically, our experimental approach, which combines different analytical methods on polymer films and injection molded specimens, identified some copolymers as best suited materials to reduce protein interaction/adsorption. Furthermore, data showed that, after the initial rapid protein adsorption (seconds/minutes), the molecular layer passivates the surface and no significant further adsorption is detected for prolonged periods, either by refrigerated storage or at room temperature.

## Figures and Tables

**Table 1:** List of different pharmaceutical grade PP materials.

n.	Manufacturer	Commercia Code	Notes
PP1	Borealis	Bormed^TM^ RD808CF	Polypropylene Random Copolymer; Melt Flow Index: 8.0 g/10 min (230 °C/2.16 kg, ISO1133); Melting temperature: 140 °C
PP2	Borealis	Bormed^TM^ SB815MO	Polypropylene Random Copolymer; Melt Flow Index: 1.5 g/10 min (230 °C/2.16 kg, ISO 1133); Melting temperature: 145 °C
PP3	Borealis	Bormed^TM^ SC876CF	Polypropylene Random Copolymer; Melt Flow Index: 3.8 g/10 min (230 °C/2.16 kg, ISO 1133); Melting temperature: 149 °C
PP4	Borealis	Bormed^TM^ HD810MO	Polypropylene Homopolymer; Melt Flow index: 10 g/10 min (230 °C/2.16 kg, ISO 1133); Melting temperature: 164 °C
PP5	LyondellBasell	Purell HP372P	Polypropylene Homopolymer; Melt Flow Index: 18 g/10 min (230 °C/2.16 kg, ISO 1133)

**Table 2:** Thicknesses of PP spin-coated films.

PP Sample	Thickness (nm)
PP1	163 ± 8
PP2	280 ± 16
PP3	200 ± 12
PP4	(40 ± 2) × 10
PP5	(40 ± 2) × 10

**Table 3:** Roughness values and QCM-D values related to rhNGF adsorption on PP substrates.

PP Sample	Ra (nm)	Δf (Hz)	ΔD (10^−6^)	Mass (ng/cm^2^)	Molecular Density (Molecules/cm^2^)
PP1	4.7 ± 1.1	−24.3 ± 0.7	0.2 ± 0.1	431 ± 13	1.0 × 10^13^
PP2	4.6 ± 0.7	−30.9 ± 0.7	0.8 ± 0.3	547 ± 12	1.2 × 10^13^
PP3	5.4 ± 1.1	−29.0 ± 0.5	0.9 ± 0.3	514 ± 9	1.2 × 10^13^
PP4	42 ± 9	−38.5 ± 1.3	0.1 ± 0.3	(68 ± 2) × 10	1.5 × 10^13^
PP5	55 ± 7	−121 ± 2	2.9 ± 1.5	(214 ± 4) × 10	4.8 × 10^13^

**Table 4:** PP thermal and wettability properties of injected molded samples.

PP Sample	Tm Onset (°C)	Tm Peak (°C)	ΔHm (J/g)	X_C_ (%)	Ra (nm)	Contact Angle (°)
PP1	120	144	61	29	8.8 ± 1.9	98 ± 2
PP2	121	151	52	25	8.3 ± 1.1	102 ± 1
PP3	127	152	40	19	7.8 ± 1.0	97 ± 2
PP4	149	165	86	41	11.5 ± 1.4	74 ± 2
PP5	148	163	84	40	12.2 ± 1.8	83 ± 1

**Table 5:** N1s/C1s intensity ratios measured on PP3 bottle after different incubation times and temperatures.

Time	N1s/C1s, 4 °C	N1s/C1s, 25 °C
30 s	0.04	0.03
7 days	0.03	0.05
30 days	0.05	0.06

## Data Availability

The data presented in this study are available on request from the corresponding author.

## Supplementary Materials

The following supporting information can be downloaded at: https://www.mdpi.com/article/10.3390/ma16052076/s1, Figure S1: D–f plot showing the kinetics of interaction of NGF with substrates. Only the third overtone is shown, due to its higher sampling depth; Table S1: Apparent diffusion coefficient and average mass transfer rate constant of NGF on different substrates; Figure S2: TM height AFM image of the inner surface of a PP3 bottle.
