# Supplementary material for: Adsorption of the rhNGF Protein on Polypropylene with Different Grades of Copolymerization
Source: Materials (Basel). 2023 Mar 3;16(5):2076. doi: 10.3390/ma16052076 (PMC10004483; doi:10.3390/ma16052076)
Supplement: Supplementary file 1 [file materials-16-02076-s001.zip › materials-2226044-supplementary.pdf]

materials

MDPI

Article

Adsorption of the rhNGF Protein on Polypropylene with Different Grades of Copolymerization

Paolo Canepa [1], Claudio Canale [1], Ornella Cavalleri [1,*], Giovanni Marletta [2], Grazia M. L. Messina [2], Massimo Messori [3], Rubina Novelli [4], Simone Luca Mattioli [5,*], Lucia Apparente [5], Nicola Detta [5], Tiziana Romeo [6] and Marcello Allegretti [6]

1 Dipartimento di Fisica, Università di Genova, Via Dodecaneso 33, 16146 Genova, Italy
2 Laboratory for Molecular Surface and Nanotechnology (LAMSUN), Dipartimento di Scienze Chimiche, Università di Catania and CSGI, Viale A. Doria 6, 95125 Catania, Italy
3 Department of Applied Science and Technology, Politecnico di Torino, Corso Duca degli Abruzzi 24, 10129 Torino, Italy
4 Research & Early Development, Dompè Farmaceutici S.p.A., Via Santa Lucia 6, 20122 Milano, Italy
5 Research & Early Development, Dompè Farmaceutici S.p.A., Via De Amicis 95, 80131 Napoli, Italy
6 Research & Early Development, Dompè Farmaceutici S.p.A., Loc. Campo di Pile, 67100 L'Aquila, Italy
* Correspondence: cavalleri@fisica.unige.it (O.C.); simone.mattioli@dompe.com (S.L.M.)

S1—Analysis of rhNGF Adsorption Kinetics by QCM-D

Figure S1 shows the D-f plots reporting the correlation between dissipation (ΔD) and frequency (Δf) values and highlighting the adsorption kinetic steps, the faster or slower kinetics corresponding to the higher or lower density of the D-f data.

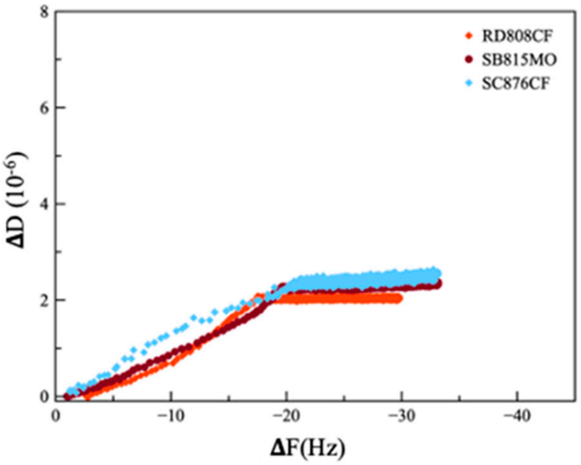

Figure S1. D–f plot showing the kinetics of interaction of rhNGF with substrates. Only the third overtone is shown, due to its higher sampling depth.

The adsorption kinetics of NGF for all the analyzed substrates are similar. The plots allow to identify two regimes, corresponding to the slope changes, which in turn can be related to different kinetics phases during the adsorption process. In fact, the first region with high slope, representing the abrupt change of frequency and dissipation (fast kinetic phase), corresponds to the early interaction events between NGF molecules and the adsorbing surface, mostly depending on the fast, transport-driven deposition and the stochastic binding of the molecules at the adsorbent surface. At variance of this, the second region of the curve shows a flat shape, suggesting the formation of a stable layer of adsorbed molecules.

A deeper analysis of the QCM-D data furthermore allows to figure out both the apparent diffusion coefficient S, describing the sticking efficiency of rhNGF on the surface, and the average mass transfer rate constant (k_c).

Indeed, the apparent diffusion coefficient (S) can be calculated from the slope of the diffusion-controlled step, by using the following relation [1]:

$$\Gamma(t) = \frac{2}{\sqrt{\pi}} c_{bulk} \sqrt{S}$$

where Γ is the adsorbed mass and c_{bulk} is the bulk concentration, while the average mass transfer rate constant (k_c) describes the efficiency of the convection-driven protein mass transport/binding at the surfaces and can be derived from the following equation [2]:

$$k_c = C_f Q^{\frac{1}{3}} S^{\frac{1}{3}}$$

where S is the apparent diffusion coefficient previously calculated, Q = 2.5 × 10^{-3} cm^3/s is the volumetric flow rate employed in the experiments and C_f = 19.9 $cm^{-4/3}$ is a geometry-dependent factor for the employed QCM-D cell. C_f is independent of the solution concentration, flow rate, and molecule size or shape.

Table S1 summarizes the parameters derived for all the analyzed substrates. Noteworthy, the S values are similar for the PP/PE copolymers, whereas its value is remarkably higher for the PP4 and PP5 homopolymers. The same trend is found for the k_c, confirming a stronger attachment of rhNGF molecules on these last two substrates.

Table S1. Apparent diffusion coefficient and average mass transfer rate constant of rhNGF on different substrates.

PP Sample	S (cm^2/s)	K_c (cm/s)
PP1	1.14 × 10^{-7}	6.36 × 10^{-5}
PP2	1.59 × 10^{-7}	7.94 × 10^{-5}
PP3	1.57 × 10^{-7}	7.88 × 10^{-5}
PP4	1.83 × 10^{-7}	8.72 × 10^{-5}
PP5	8.42 × 10^{-7}	2.41 × 10^{-4}

S2—AFM Analysis on Injection Moulded PP3 Bottles

The roughness of the inner surface of PP3 bottles has been evaluated by AFM, using the same acquisition mode described in the Materials and Methods section.

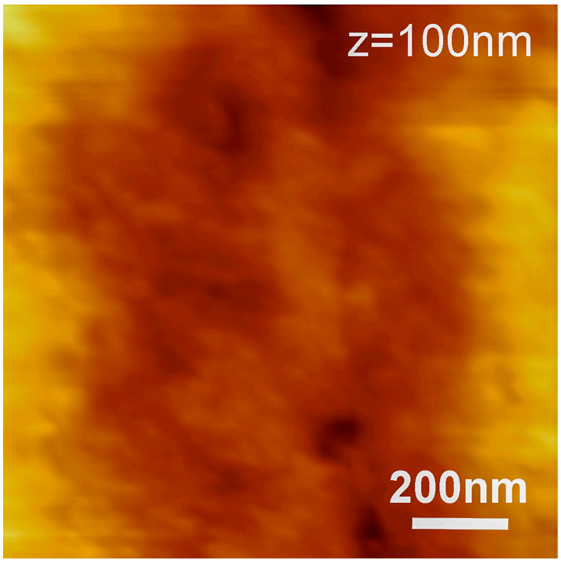

Figure S2. TM height AFM image of the inner surface of a PP3 bottle.

Figure S2 shows the typical morphology of the inner surface of PP3 bottles, with a mean Ra value of (15 ± 4) nm.

References

1. Almeida, A.T.; Salvadori, M.C.; Petri, D.F.S. Enolase Adsorption onto Hydrophobic and Hydrophilic Solid Substrates. *Langmuir* **2002**, *18*, 6914–6920, doi:10.1021/la0202982.
2. Kubiak, K.; Adamczyk, Z.; Oćwieja, M. Kinetics of Silver Nanoparticle Deposition at PAH Monolayers: Reference QCM Results. *Langmuir* **2015**, *31*, 2988–2996, doi:10.1021/la504975z.
